# Novel Remineralizing and Antibiofilm Low-Shrinkage-Stress Nanocomposites to Inhibit Salivary Biofilms and Protect Tooth Structures

**DOI:** 10.3390/ma16206770

**Published:** 2023-10-19

**Authors:** Abdullah Alhussein, Rashed Alsahafi, Areej Alfaifi, Mohammad Alenizy, Ibrahim Ba-Armah, Abraham Schneider, Mary-Ann Jabra-Rizk, Radi Masri, Guadalupe Garcia Fay, Thomas W. Oates, Jirun Sun, Michael D. Weir, Hockin H. K. Xu

**Affiliations:** 1 PhD Program in Dental Biomedical Sciences, University of Maryland School of Dentistry, Baltimore, MD 21201, USA; aalhussein@ksu.edu.sa (A.A.);; 2 Department of Restorative Dental Sciences, College of Dentistry, King Saud University, Riyadh 11451, Saudi Arabia; 3 Department of Restorative Dental Sciences, Umm Al-Qura University, College of Dentistry, Makkah 24211, Saudi Arabia; 4 Department of Restorative and Prosthetic Dental Sciences, College of Dentistry King Saud bin Abdulaziz University for Health Sciences, Riyadh 14611, Saudi Arabia; 5 Department of Oncology and Diagnostic Sciences, University of Maryland School of Dentistry, Baltimore, MD 21201, USA; 6 Biomaterials & Tissue Engineering Division, Department of Advanced Oral Sciences and Therapeutics, University Maryland School of Dentistry, Baltimore, MD 21201, USA; 7 The Forsyth Institute, Harvard School of Dental Medicine Affiliate, Cambridge, MA 02142, USA; 8 Center for Stem Cell Biology & Regenerative Medicine, University of Maryland School of Medicine, Baltimore, MD 21201, USA; 9 Marlene and Stewart Greenebaum Cancer Center, University of Maryland School of Medicine, Baltimore, MD 21201, USA

**Keywords:** remineralizing, calcium fluoride nanoparticles, dimethylaminododecyl methacrylate, amorphous calcium phosphate nanoparticles, antibiofilm, caries

## Abstract

Recurrent caries remain a persistent concern, often linked to microleakage and a lack of bioactivity in contemporary dental composites. Our study aims to address this issue by developing a low-shrinkage-stress nanocomposite with antibiofilm and remineralization capabilities, thus countering the progression of recurrent caries. In the present study, we formulated low-shrinkage-stress nanocomposites by combining triethylene glycol divinylbenzyl ether and urethane dimethacrylate, incorporating dimethylaminododecyl methacrylate (DMADDM), along with nanoparticles of calcium fluoride (nCaF_2_) and nanoparticles of amorphous calcium phosphate (NACP). The biofilm viability, biofilm metabolic activity, lactic acid production, and ion release were evaluated. The novel formulations containing 3% DMADDM exhibited a potent antibiofilm activity, exhibiting a 4-log reduction in the human salivary biofilm CFUs compared to controls (*p* < 0.001). Additionally, significant reductions were observed in biofilm biomass and lactic acid (*p* < 0.05). By integrating both 10% NACP and 10% nCaF_2_ into one formulation, efficient ion release was achieved, yielding concentrations of 3.02 ± 0.21 mmol/L for Ca, 0.5 ± 0.05 mmol/L for P, and 0.37 ± 0.01 mmol/L for F ions. The innovative mixture of DMADDM, NACP, and nCaF_2_ displayed strong antibiofilm effects on salivary biofilm while concomitantly releasing a significant amount of remineralizing ions. This nanocomposite is a promising dental material with antibiofilm and remineralization capacities, with the potential to reduce polymerization-related microleakage and recurrent caries.

## 1. Introduction

Recurrent dental caries continue to be a major contributor to the significant expenses associated with repeat dental treatments [1,2,3]. Dental plaque consists of multispecies biofilms that form on tooth surfaces [4,5]. Among these species, *Streptococcus mutans* (*S. mutans*) is a significant contributor to dental caries [6,7]. The cariogenic potential of *S. mutans* arises from three primary factors, including the production of extracellular glucan polymers from sucrose, metabolizing carbohydrates into acids, and the ability to thrive in low pH conditions [8,9]. In addition, oral biofilms play a role in the dissolution of tooth minerals by producing organic acids [10,11]. Therefore, the development of dental restorations with improved marginal integrity and bioactivity, such as antibacterial and remineralization capabilities, is beneficial. This achievement could enhance the clinical durability of dental restorations and improve the cost-effectiveness of oral healthcare [12,13]. Several studies have investigated efforts to improve marginal integrity through a reduction in polymerization shrinkage stress [14,15]. Previous investigations have explored the development of resin systems with distinctive polymerization characteristics aimed at a reduction in polymerization shrinkage and shrinkage stress [16,17,18]. These include thiolene resin-base, epoxy resin-base, and silorane resin-base [16,17,18]. Recently, a novel slow polymerization rate resin composite was developed using urethane dimethacrylate (UDMA) and an ether-based triethylene glycol divinylbenzyl ether (TEG-DVBE) [19]. This formulation successfully reduced polymerization shrinkage stress by 47% compared to traditional composites [15]. Moreover, this formulation incorporates TEG-DVBE as a diluted monomer, providing resistance against esterase and hydrolytic degradation [19]. In a previous study, this innovative resin system was used to develop a dental adhesive [19]. It demonstrated improved resistance to hydrolytic challenges and achieved excellent microtensile bond strength after thermocycling compared with a commercial adhesive resin system [19].

Managing the development of recurrent dental caries should involve considering it as a condition that depends on the presence of biofilms [20,21]. Previously, several research studies have integrated different antibiofilm substances into dental resins to inhibit the formation and metabolic activity of biofilms, including quaternary ammonium methacrylates (QAMs) [22,23]. These investigations demonstrated that incorporating QAMs into dental resins could enhance the antibiofilm effectiveness without adversely affecting the mechanical and physical properties [15]. For instance, previous studies have focused on the development of an antibiofilm resin-based composite by incorporating ionic dimethacrylate monomers (IDMAs) [22]. This approach aimed to inhibit bacterial growth without causing any cytotoxic effects [22]. Furthermore, other approaches have integrated various QAM monomers into their investigations, such as quaternary ammonium polyethylenimine (QPEI) [24] and 12-methacryloyloxydodecylpyridinium bromide (MDPB) nanoparticles [25]. In recent studies, the addition of dimethylaminododecyl methacrylate (DMADDM) as an antibiofilm agent in resin-based restorative materials has shown promising results [15,26]. DMADDM, characterized by its long chain length, exhibits heightened hydrophobic properties, resulting in a greater degree of hydrophobicity that contributes to its enhanced antibiofilm potency [27]. This is attributed to its extended molecular structure, which not only increases its hydrophobic nature but also results in higher surface charge densities [27]. Furthermore, DMADDM has the capacity to copolymerize with traditional dental resin monomers and provide a potent antibiofilm effect through a contact-killing mechanism [28]. In our recent investigation, we successfully developed a novel antibiofilm composite material by incorporating up to 5% DMADDM into a novel formulated low-shrinkage-stress resin [15]. The results were highly promising, showing a significant 46% reduction in shrinkage stress compared to the traditional composite [15]. Importantly, these improvements were achieved without compromising the mechanical properties of the composite material [15].

An alternative approach to preventing the development of recurrent caries involves incorporating remineralization filler, such as nano-fluorapatite, nano-hydroxyapatite, nano-sized calcium fluoride (nCaF_2_) or nano-sized amorphous calcium phosphate (NACP), into resin-based materials [29,30,31]. These fillers provide a practical method for introducing bioactivity. By adding them to composites, the process of remineralization/demineralization can be influenced through the controlled release of a clinically relevant concentration of remineralizing ions [32,33]. nCaF_2_ is particularly effective at releasing calcium (Ca) and fluoride (F) ions, which can aid in reducing bacterial acid production and facilitating remineralization [32,34]. Additionally, NACP has been shown to enhance the remineralization of tooth structure [33]. This approach holds promise in reducing demineralization and improving the longevity of resin composites. Our recent investigation aimed to develop a bioactive low-shrinkage-stress nanocomposite containing 3% DMADDM, along with either nCaF_2_ or NACP [31]. These novel formulations exhibited potent antibiofilm effects without compromising the mechanical properties and cell viability of dental pulp stem cells and human gingival fibroblasts [31]. However, the antibiofilm efficacy of these formulations was assessed against an *S. mutans* biofilm [15,31]. However, to date, there has been no report on the release of ions and the antibiofilm properties against complex biofilm models when incorporating DMADDM with NACP and nCaF_2_ in a low-shrinkage-stress nanocomposite.

Therefore, the current study serves as a continuation of our earlier research, focusing on the performance of a low-shrinkage-stress nanocomposite with remineralizing and antibiofilm capabilities. The objective of this study was to investigate the ion release and antibiofilm effectiveness against multispecies human salivary biofilms for our novel formulations. The following hypotheses were tested: (1) The novel low-shrinkage-stress nanocomposite incorporating DMADDM with nCaF_2_ and NACP could greatly reduce bacterial viability, metabolic activity, and lactic acid production, (2) The combination of DMADDM with nCaF_2_ and NACP could provide the release of high levels of Ca, P and F ions.

## 2. Materials and Methods

### 2.1. Synthesizing a Nanocomposite Comprising Dimethylaminododecyl Methacrylate, Amorphous Calcium Phosphate, and Calcium Fluoride Nanoparticles

The experimental composite was formulated using a combination of 44.2% TEG-DVBE and 55.8% UDMA (all mass %), denoted as UV, as previously reported [13,15]. The low-shrinkage-stress composite was photoactivated with 0.8% ethyl 4-(dimethylamino) benzoate (4EDMAB; Millipore Sigma, Burlington, MA, USA) and 0.2% camphorquinone (CQ, Millipore Sigma). For the synthesis of DMADDM, a modified Menschutkin reaction was utilized, as described in previous studies [15,35]. Subsequently, DMADDM was introduced into our formulations, comprising a final mass fraction of 3%.

nCaF_2_ (particle size = 32 nm) and NACP (particle size = 116 nm) nanoparticles were synthesized utilizing a spray-drying process, as described in previous studies [31,32]. To ensure strong mechanical characteristics and promote remineralization, nCaF_2_, and NACP fillers were added to the final novel composite at a mass fraction of 20% [31]. Additionally, silanized barium boroaluminosilicate glass particles (Dentsply Sirona, Milford, DE, USA) were incorporated at 45% for mechanical reinforcement. Heliomolar (Ivoclar, ON, Canada), comprising 66.7% nano-filled particles of silica and ytterbium-trifluoride, was selected as a commercial control composite, which is known to release fluoride as a comparison [15]. The choice of a 65% glass filler level was based on our earlier investigation to formulate a bioactive low-shrinkage-stress nanocomposite with excellent mechanical properties [31].

Five composite groups were assessed as listed below:

1. Heliomolar resin composite (designated as “Commercial Control Composite”);
2. Experimental resin composite: 35% UV + 65% glass (designated as “Experimental Control Composite”);
3. 32% UV + 3% DMADDM + 20% NACP + 45% glass (designated as “NACP+DMADDM Nanocomposite”);
4. 32% UV + 3% DMADDM + 20% nCaF_2_ + 45% glass (designated as “nCaF_2_+DMADDM Nanocomposite”);
5. 32% UV + 3% DMADDM + 10% NACP + 10% nCaF_2_ + 45% glass (designated as “NACP+nCaF_2_+DMADDM Nanocomposite”).

### 2.2. Samples Preparation for Biofilm Testing

Composite specimens (d = 9 mm, t = 2 mm) were prepared and photo-cured for 60 s (Labolight DUO, GC America, Alsip, IL, USA) and incubated in a 37 °C incubator for 24 h [15]. Eliminating the residual monomer was achieved via 1 h agitation in distilled water [15]. Specimens were sterilized with ethylene oxide (Anprolene AN 74i, Andersen, Haw River, NC, USA). In accordance with the manufacturer’s instructions, we implemented a seven-day degassing period to eliminate any residual ethylene oxide from the samples.

### 2.3. Human Saliva-Based Microcosm Biofilm Model

In this study, a biofilm model was developed using human saliva, following the methods described in previous research [34]. Saliva collection adhered to the principles of the Declaration of Helsinki and received approval from the University of Maryland Baltimore’s Institutional Review Board (IRB#: HP-00050407). Saliva was collected from ten healthy individuals with no active caries. Donors were instructed to refrain from brushing their teeth for 24 h and avoid consuming food and drinks for 2 h before collection. Saliva was diluted to a 70% concentration with glycerol and stored at −80 °C [34]. The saliva–glycerol solution was mixed with the McBain artificial saliva growth medium at a final dilution of 1:50, supplemented with 0.2% sucrose. Composite samples were immersed in 1.5 mL of the inoculum within 24-well plates, followed by incubation at 37 °C in a 5% CO_2_ environment. Fresh medium was supplemented at 8 and 24 h intervals during incubation. Based on a previous study, a total incubation time of 48 h was found to be sufficient for the formation of mature dental plaque microcosm biofilms on composite disks [34].

### 2.4. Visualization of Live/Dead Staining of Biofilms

After 48 h of incubation, the composite samples with attached biofilms were washed three times with 1 mL of phosphate-buffered saline (PBS) for 10 s. The composite samples (*n* = 3) were stained using the BacLight live/dead kit (Molecular Probes, Eugene, OR, USA) in accordance with the manufacturer’s guidelines. A combination of 2.5 μM SYTO 9 and 2.5 μM propidium iodide was used to stain each sample for 15 min. This technique facilitated the distinction between live bacteria, exhibiting green fluorescence upon SYTO 9 staining, and bacteria with damaged cell membranes producing red fluorescence following propidium iodide staining. Fluorescently stained biofilms were visualized using an epifluorescence microscope (Eclipse TE2000-S, Nikon, Melville, NY, USA) [34].

### 2.5. Biofilm Colony Forming Units (CFU) Count

After 48 h of incubation, the composite samples (*n* = 6) with attached biofilms were washed three times in 1 mL of cysteine peptone water (CPW) for 10 s. Subsequently, all samples were transferred to a vial containing 1 mL of CPW and subjected to sonication for 7 min, followed by vortexing for 5 s to harvest the biofilm [34]. The bacterial suspensions were then serially diluted (by 10^1^ to 10^6^ folds) and dropped onto agar plates. These agar plates were incubated at 37 °C in a 5% CO_2_ environment for 48 h. After the incubation period, the colony number was counted, and the biofilm colony-forming units (CFU) were determined [34]. The CFU experiment was conducted in triplicate. Three different agar plates were utilized for the CFU experiments:

1. Tryptic soy blood agar (TSA) supplemented with defibrinated sheeps blood was used to assess the biofilm growth of total microorganisms.
2. Mitis salivarius agar (MSA) was employed to evaluate the biofilm growth of total Streptococci.
3. MSA with 0.2 units of bacitracin per mL and potassium tellurite (Millipore Sigma) (MSB) was used to assess the biofilm growth of Mutans streptococci.

### 2.6. Metabolic Activity Exhibited by Biofilms

The biofilm’s metabolic activity was evaluated using a 3-[4,5-dimethylthiazol-2-yl]-2,5-diphenyltetrazolium bromide (MTT) colorimetric assay [31]. Mature biofilm-coated composites (*n* = 6) were placed in a 24-well plate containing 1 mL of MTT dye and incubated at 37 °C with 5% CO_2_ for 1 h. Following this, the samples were immersed in 1 mL of dimethyl sulfoxide (DMSO) for 20 minutes to dissolve the formazan crystals. Subsequently, 200 μL of the DMSO solution from each specimen was transferred to a 96-well plate for absorbance measurement. The absorbance was assessed at 540 nm using a microplate reader (SpectraMax M5, Molecular Devices L.L.C., San Jose, CA, USA). Higher absorbance values indicated a greater formazan concentration, indicating increased biofilm metabolic activity on the disk [31]. The metabolic activity experiment was conducted in triplicate.

### 2.7. Production of Lactic Acid by Biofilms

After incubating the composite samples (*n* = 6) with attached biofilms for 48 h, the composite samples were immersed in 1.5 ml of buffered peptone water (B.P.W., Aldrich, St. Louis, MO, USA) and supplemented with 0.2% sucrose. The plates were incubated for a period of 3 h at a temperature of 37 °C with 5% CO_2_ [22]. We measured the optical density at a wavelength of 340 nm using a microplate reader (SpectraMax M5, Molecular Devices, Sunnyvale, CA, USA) to determine the lactate concentrations in the BPW. This measurement was performed using an enzymatic assay based on lactate dehydrogenase, following well-established procedures [31]. The lactic acid production experiment was conducted in triplicate.

### 2.8. Assessment of the Release of Calcium, Phosphate, and Fluoride Ions

The composite specimens (*n* = 3) were immersed in a pH 4 solution with a volume of 50 mL to simulate the cariogenic conditions in the oral cavity, as described in a previous study [26]. Each group had three tubes, and all tubes were stored at 37 °C throughout the experiment.

The ion release from the specimens was measured at 1, 3, 5, 7, 14, 21, 28, 35, 42, 49, 56, 63, and 70 days. During each designated time interval, 2 mL from each tube was extracted and subsequently refilled with a fresh solution. Throughout the experiment, the immersion solution was kept at a pH of 4.

The concentrations of Ca and P ions were analyzed using the spectrophotometric method (SpectraMax M5) [34]. For the measurement of F ion release, a fluoride ion selective electrode (Orion, Cambridge, MA, USA) was used. To determine the F ion concentration in the samples, 0.5 mL of the sample was combined with 0.5 mL of the undiluted TISAB solution (Fisher Scientific, Pittsburgh, PA, USA).

#### Statistical Analysis

Sigma Plot (SYSTAT, Chicago, IL, USA) was used for statistical analyses. The significant differences were texted using one-way analyses of variance (ANOVA) and Tukey’s comparison. Results were considered statistically significant at a *p* < 0.05.

## 3. Results

### 3.1. Live/Dead Staining of Salivary Biofilms

**Figure 1:**
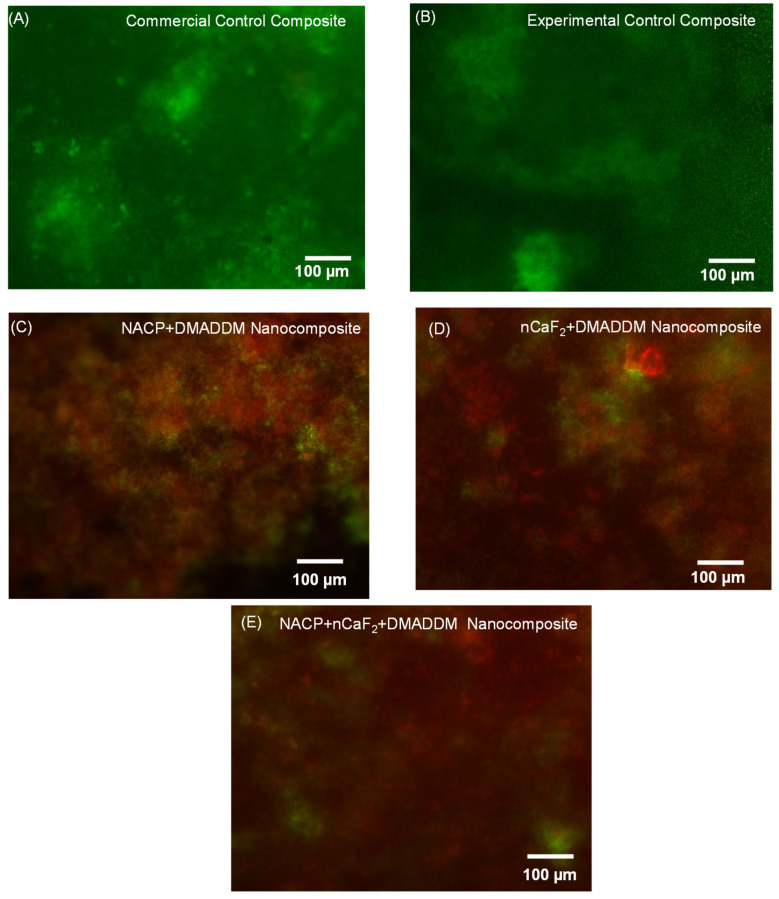
Live/dead staining of salivary biofilms on composite disks. (**A**) Commercial control composite. (**B**) Experimental control composite. (**C**) DMADDM+NACP nanocomposite. (**D**) DMADDM+nCaF_2_ nanocomposite. (**E**) DMADDM+NACP+nCaF_2_ nanocomposite. The commercial and experimental control composites were observed to have live bacteria covering their surfaces, indicated by the green stain. However, in contrast, when DMADDM was added with NACP, nCaF_2_, or both into the low-shrinkage-stress nanocomposite, a higher number of dead bacteria were observed, as evidenced by the red stain.

Figure 1 depicts the biofilm-covered composite surface at 48 h. The control groups’ surface showed salivary biofilms predominantly consisting of live bacteria. However, the nanocomposites incorporating DMADDM with NACP, nCaF_2_, or both inhibited salivary biofilm growth, as indicated by red staining.

### 3.2. Biofilm Colony-Forming Units Counts

**Figure 2:**
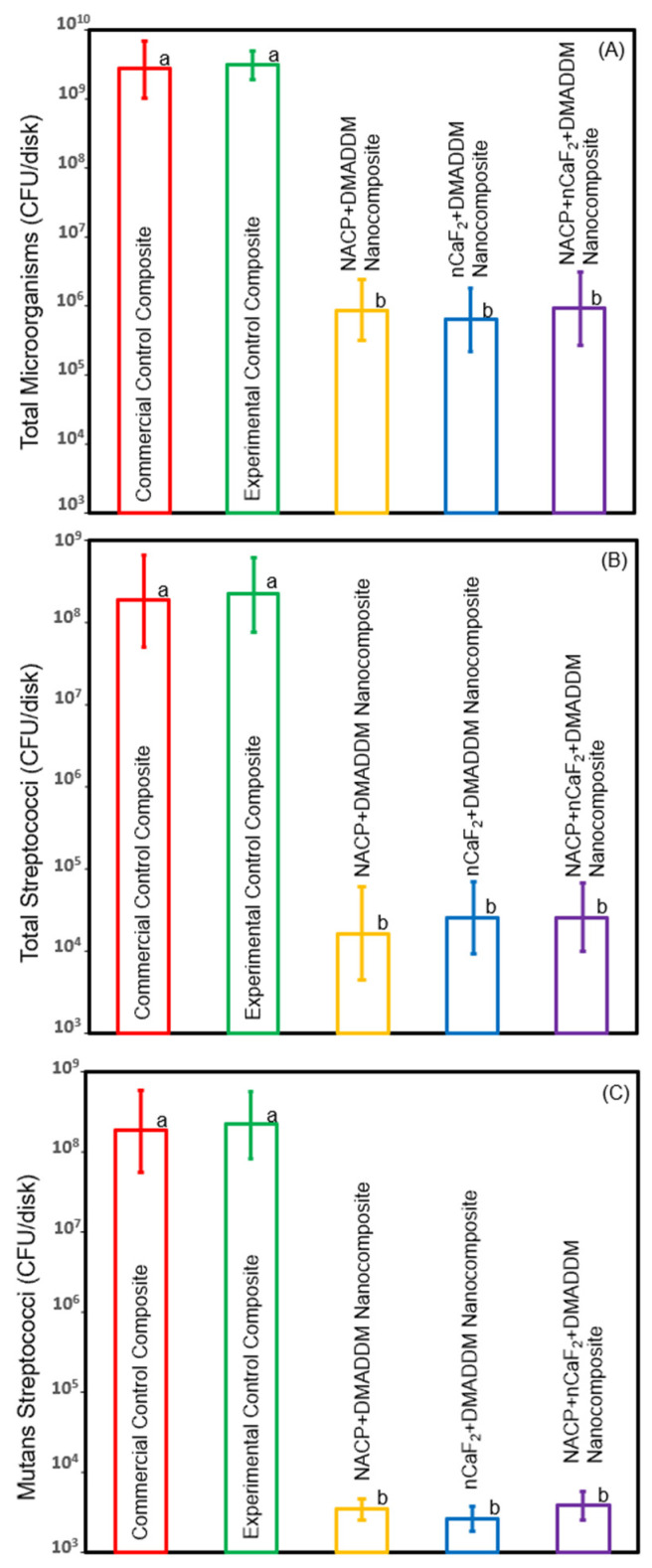
Biofilm colony-forming unit (CFU) counts on composite samples (mean±sd; n=6). (**A**) Total microorganisms (TSA); (**B**) Total streptococci (MSA), and (**C**) Mutans streptococci (MSB). The control groups demonstrated the most extensive biofilm growth. The innovative formulations, containing DMADDM with NACP, nCaF_2_, or both, significantly reduced salivary biofilm development compared to the commercial control (*p* < 0.05). These formulations substantially decreased salivary biofilm growth by 3–4 orders of magnitude compared to controls (*p* < 0.05). Dissimilar letters indicate values that are significantly different from each other (*p* < 0.05).

Figure 2 illustrates the CFU of salivary biofilm (mean±sd; n=6). The nanocomposites, incorporating DMADDM with NACP, nCaF_2_, or both, demonstrated a significant reduction in CFU counts of total microorganisms when compared to the control groups (*p* < 0.05). Furthermore, the antibiofilm low-shrinkage-stress nanocomposites were particularly effective against total streptococci and mutans streptococci, achieving a 4-log reduction compared to the controls (*p* < 0.05).

### 3.3. MTT Assay of Metabolic Activity of Salivary Biofilms

**Figure 3:**
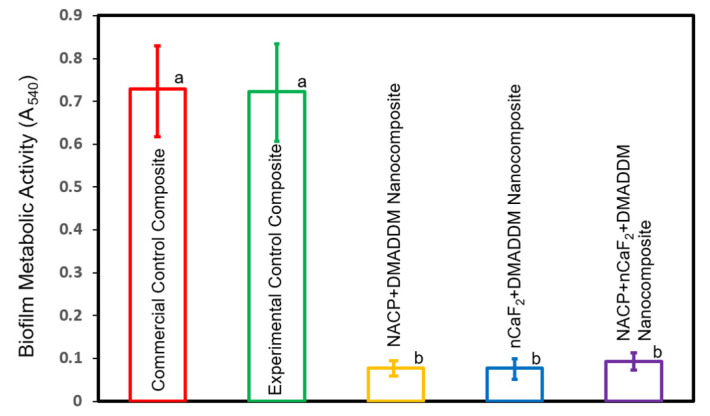
Metabolic activity of biofilms using the MTT assay (mean±sd; n=6). The experimental formulations containing DMADDM with NACP, nCaF_2_, or both achieved a significant reduction in the metabolic activity of salivary biofilms compared to the controls (*p* < 0.05). Different letters represent values that show significant differences from each other (*p* < 0.05).

Figure 3 displays the metabolic activity of 48 h biofilms on the composites surface. The composite containing DMADDM with NACP, nCaF_2_, or both, caused a significant reduction in the metabolic activities of salivary biofilm compared to the controls (*p* < 0.05). However, there were no significant differences in the metabolic activities observed among the DMADDM-containing groups (*p* > 0.05).

### 3.4. Lactic Acid Production by Salivary Biofilms

**Figure 4:**
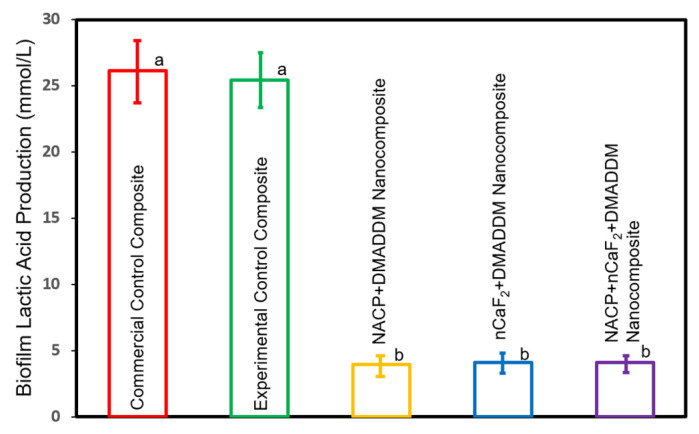
Lactic acid production by salivary biofilms on the composite surface (mean±sd; n=6). The lactic acid concentration was highest in the control groups (*p* < 0.05). However, the formulations containing DMADDM with NACP, nCaF_2,_ or both significantly reduced lactic acid production compared to the other groups (*p* < 0.05). Different letters represent values that show significant differences from each other (*p* < 0.05).

Figure 4 displays the lactic acid production of salivary biofilms formed on the composite surface $\left(\mathrm{mean}\pm \mathrm{sd};\;n=6\right)$. The acid production was highest in the control groups (*p* < 0.5). However, the innovative formulations containing DMADDM with NACP, nCaF_2_, or both significantly reduced the acid concentration compared to the commercial group (*p* < 0.05). Nevertheless, there were no significant differences in acid production among these DMADDM-containing groups (*p* > 0.05).

### 3.5. Ions Release for Calcium, Phosphate, and Fluoride

**Figure 5:**
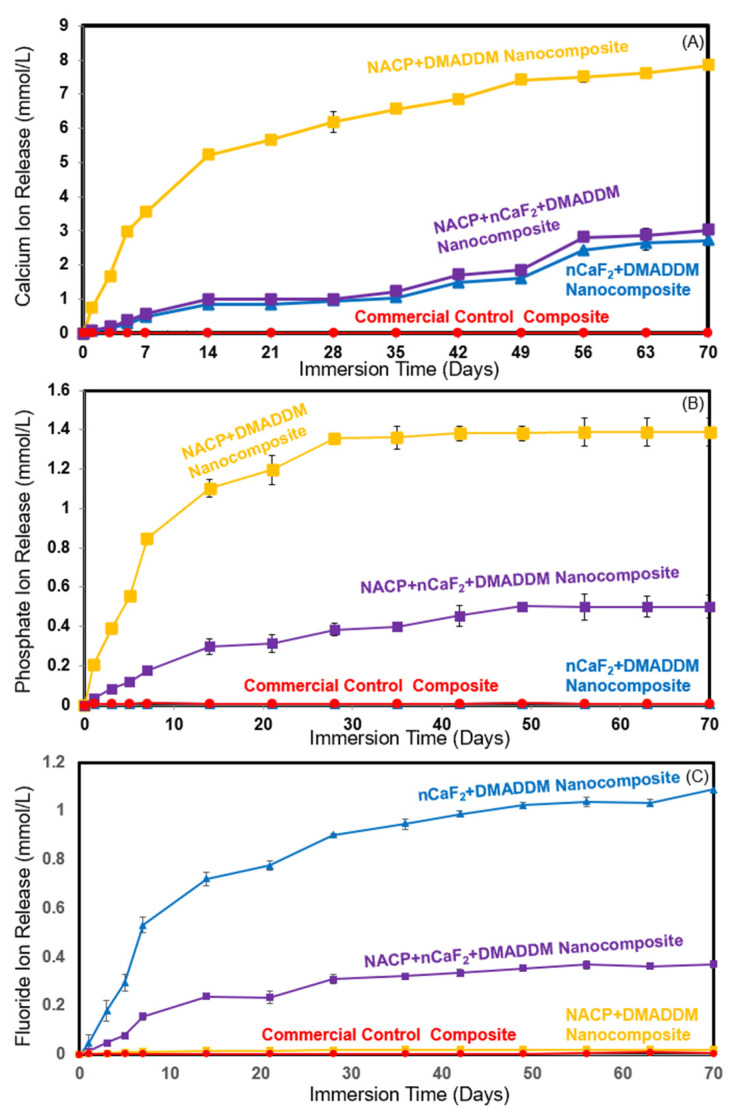
The release of calcium, phosphate, and fluoride ions from nanocomposite specimens (mean ± sd; *n* = 6): (**A**) Calcium ion release, (**B**) Phosphate ion release, and (**C**) Fluoride ion release. The innovative nanocomposite, incorporating 10% NACP and 10% nCaF_2_, demonstrated dual nanofiller benefits by releasing substantial Ca, P, and F ions.

The Ca, P, and F ions released from nanocomposite specimens are plotted in Figure 5. For Ca ion release, at 70 days, the nanocomposite with 20% NACP had the highest Ca ion release at (7.84 ± 0.08) mmol/L (*p* < 0.05), followed by the nanocomposite with both 10% NACP and 10% nCaF_2_ (3.02 ± 0.21) mmol/L (*p* < 0.05) and the nanocomposite with 20% nCaF_2_ (2.7 ± 0.15) mmol/L (*p* < 0.05). The commercial control composite demonstrated no release of Ca ions. For P ion release, among all the composite groups, the addition of 20% NACP in the nanocomposite resulted in the highest P ion release at (1.30 ± 0.07) mmol/L (*p* < 0.05), followed by the nanocomposite containing 10% NACP and 10% nCaF_2_ (0.5 ± 0.05) mmol/L (*p* < 0.05). The nanocomposite with 20% nCaF_2_ and a commercial control composite revealed the lowest results with the close to zero release of the P ion at (0.005 ± 0.001) mmol/L and (0.005 ± 0.001) mmol/L, respectively. (*p* < 0.05). For F ion release, the nanocomposite with 20% nCaF_2_ had the highest F ion release at (1.09 ± 0.01) mmol/L, and the nanocomposite with both 10% NACP and 10% nCaF_2_ had an F ion release of (0.37 ± 0.01) mmol/L (*p* < 0.05). However, the commercial control and nanocomposite with 20% NACP revealed a limited F ion release at (0.006 ± 0.001) mmol/L and (0.019 ± 0.003) mmol/L, respectively (*p* < 0.05).

## 4. Discussion

This study investigated the ion release and antibiofilm effects against human salivary biofilm for a low-shrinkage-stress nanocomposite incorporating DMADDM, NACP, and nCaF_2_ for the first time. The integration of these components in a low-shrinkage-stress nanocomposite offers potential benefits such as reducing polymerization shrinkage stress, preventing secondary caries, and preserving tooth structure. This outcome could improve the durability of composite restorations. In the current study, our investigation focused on two main objectives. The first objective was to assess the capacity of the bioactive low-shrinkage-stress nanocomposite to inhibit multi-species high-challenge biofilms resembling the oral ecosystem. The second objective was to evaluate the ion release from these formulations. This study successfully demonstrated the capability of the novel bioactive low-shrinkage-stress nanocomposite to inhibit human salivary microcosm biofilm growth and achieve high levels of ion release, thereby confirming this study’s hypotheses. Specifically, the addition of DMADDM with NACP and nCaF_2_ into a low-shrinkage nanocomposite resulted in a significant 4-log reduction in human salivary biofilm growth. Furthermore, these novel formulations demonstrated a significant decrease of over 84% in the production of lactic acid and metabolic activity within the human salivary microcosm biofilm. This finding highlights the potent antibiofilm effect of the material while also maintaining high levels of ion release. Moreover, the identical composition employed in this investigation previously exhibited excellent mechanical characteristics and compatibility with both human gingival fibroblasts and dental pulp stem cells [31]. In addition, these novel formulations demonstrated potent antibiofilm properties against *S. mutans* biofilm [31]. However, it is essential to highlight that no previous study has explored the effectiveness of these novel formulations against a multi-species human salivary biofilm model, which was performed in the present study for the first time.

Resin composite restorations have been associated with a relatively high failure rate, primarily due to tooth fractures and recurrent caries [36,37]. These factors can pose significant challenges in maintaining the longevity and effectiveness of dental composite restorations [36]. These issues arise due to the material’s lack of bioactivity, and the development of polymerization shrinkage stresses at the tooth–restoration interface [36,38]. As polymerization takes place, the initiation of shrinkage stress leads to composite contraction and decreased flowability, resulting in residual stresses arising from bonding constraints between the composite material and tooth structures [38,39,40,41]. These marginal gaps may facilitate recurrent caries, especially when resin-based dental materials lack bioactivity [15,42]. Addressing these factors is crucial to improving the durability and longevity of resin composite restorations. In the past, several attempts have been made to develop composite materials with low volumetric shrinkage or low shrinkage stress [16,17,18,43,44]. However, these endeavors have faced challenges, resulting in compromising mechanical properties with limited improvements in marginal integrity [45,46,47]. Nonetheless, a recent breakthrough emerged with the introduction of a novel low-shrinkage-stress composite that utilized TEGDVBE and UDMA monomers [19]. This innovative approach resulted in a reduced polymerization rate, allowing for stress relaxation and preventing excessive contraction stresses [19]. Moreover, this formulation enhanced the resistance of the low-shrinkage-stress resin-base material against salivary hydrolysis, hydrolytic challenges, and esterase degradation [19]. Additionally, this innovative formulation minimized polymerization stress, improved mechanical strength, and exhibited a high degree of conversion [15,19]. This advancement shows great promise in overcoming previous limitations in dental restorative materials. Our previous investigation showed a substantial 46% reduction in polymerization shrinkage stresses in a low-shrinkage-stress composite containing DMADDM compared to a conventional resin-based control composite [15]. Nevertheless, there has been no report that explored the antibiofilm properties and ion release resulting from the combination of DMADDM with nCaF_2_ and NACP within a low-shrinkage-stress nanocomposite when exposed to a more clinically relevant human salivary microcosm biofilm.

Dental plaque exerts a significant impact on the longevity of dental restorations, giving rise to various issues like cariogenic bacterial attachment, material degradation, and recurrent caries [48,49,50]. Addressing this concern, the exploration of innovative bioactive low-shrinkage-stress materials has become imperative. These materials possess antibiofilm and remineralization properties that could enhance the durability and longevity of composite restorations. Previous attempts aimed to develop antibiofilm resin composites through the incorporation of agents such as chlorhexidine, silver, and zinc oxide nanoparticles [33,51]. However, these attempts have shown short-term effects, often at the expense of mechanical properties [33,51]. To overcome this critical drawback, a novel approach involving quaternary ammonium monomers (QAMs) has been developed [27]. These QAMs can be covalently bonded with dental resins, providing long-term antibiofilm effects [27]. Incorporating QAMs in methacrylate-based materials has demonstrated potent antibiofilm effects through a contact-killing mechanism [28,52]. The mode of action for QAMs involves interactions between the positively charged quaternary amine N^+^ and the negatively charged components of the bacterial cell membrane [28]. This interaction disrupts the electron balance at the bacterial cell membrane, subsequently leading to an elevation in osmotic pressure [28]. Ultimately, this increased osmotic pressure results in the rupture of the cell membrane and subsequent bacterial cell death [28]. Among these QAMs, the DMADDM monomer has shown significant antibiofilm effects without compromising mechanical properties [15,27]. Furthermore, a previous investigation showed a durable antibiofilm activity of resin-base material containing DMADDM after 6 months of water aging without compromising mechanical properties [35]. Another previous study determined the optimal concentration of DMADDM and showed that strong antibiofilm effects were achieved with 3% DMADDM in a low-shrinkage-stress composite without significantly reducing the composite’s mechanical properties [15]. In our recent investigation, we observed that the addition of 20% NACP or 20% nCaF_2_ into a resin-based composite demonstrated no significant antibiofilm effect [31]. By contrast, the incorporation of 3% DMADDM with either 20% NACP or 20% nCaF_2_ exhibited a potent antibiofilm effect against *S. mutans* biofilms by reducing the CFU count by six logs, when compared to the control [31]. However, previous investigations demonstrated that introducing DMADDM into conventional resin-based material aimed at combating salivary biofilms led to an approximate two-log reduction [26]. This reduction in antibiofilm effectiveness could be due to the increase in multispecies biofilms resistant to antibiofilm treatments compared to biofilms established by a single strain. In this study, our novel formulations achieved a 3- to 4-log reduction in the CFU count of saliva-derived biofilms compared to the control groups (*p* < 0.05). Our novel antibiofilm nanocomposites demonstrated a substantial four-log reduction in both total Streptococci and Mutans Streptococci, compared to incorporating DMADDM into a traditional resin [26]. This outcome suggests that the slower polymerization rate of the UV resin may have contributed to the more even distribution of DMADDM through the resin, potentially enhancing its antibacterial effectiveness. Furthermore, our formulations exhibited a comparable result when compared to the commercially available Clearfil Protect Bond, which contains MDPB as an antibiofilm agent [53]. Further studies are needed to investigate the effects of different matrix resin compositions on antibacterial properties. Furthermore, the images depicting live and dead bacteria offer further proof of the antibiofilm characteristics exhibited by our novel formulations, which showed more dead bacteria present on the surface compared with the control groups. In addition, these novel formulations containing DMADDM achieved an 84% reduction in lactic acid production and metabolic activity of saliva-derived biofilms (*p* < 0.05). However, our results showed no significant difference in antibiofilm effect when incorporating DMADDM with either NACP, nCaF_2_, or combined against saliva-derived biofilms (*p* > 0.05).

The presence of dental plaque can lead to the production of acidic attacks, disturbing the dynamic equilibrium of minerals and substances within the saliva and tooth structure [54,55,56]. Therefore, developing an innovative formulation with antibiofilm and remineralization properties might effectively impede recurrent caries by diminishing biofilm formation and releasing ions such as Ca, P, and F. These ions could facilitate the process of remineralizing the demineralized tooth structure [57,58,59]. A previous investigation revealed that the presence of Ca, P, and F ions plays a role in the creation of fluoridated hydroxyapatite, enhancing its resistance against acid attacks [57]. Moreover, the presence of F ions can expedite the precipitation of Ca and P, which facilitates the formation of hydroxyapatite crystals during remineralization [60,61]. This process could result in the development of larger crystals that are more resistant to acid attacks [60,62]. A promising approach to preventing recurrent caries may be achieved through the incorporation of remineralization fillers like NACP and nCaF_2_ into composite restorations. These fillers possess smaller particle sizes, resulting in a larger surface area that can facilitate a higher level of ion release [23,33]. Furthermore, previous investigations have demonstrated that incorporating 20% NACP into a low-shrinkage-stress composite does not compromise the mechanical properties even after 20,000 thermocycles when compared to commercial composite [13]. Another study examined the mechanical properties of a composite containing 20% nCaF_2_ after 100,000 thermal cycles, revealing excellent mechanical performance [32]. In the present study, the novel nanocomposite containing 20% NACP resulted in the most substantial release of Ca and P ions. However, this formulation did not exhibit a notable release of F ions. Conversely, the integration of 20% nCaF_2_ displayed the capacity to release a substantial amount of F ions. However, it exhibited a lower level of P ion release. The innovative formulation, containing DMADDM with both NACP and nCaF_2_, showcased a blend of benefits from these two types of nanofillers. This composite demonstrated the capability to release significant quantities of Ca, P, and F ions. The ion release from this formulation exceeded the concentration of ions required for promoting tooth remineralization, as reported by previous studies [63]. They found that remineralization could be achieved with as little as 0.30 mmol/L of Ca and 0.015 mmol/L of F ions [63].

This novel approach of incorporating DMADDM with both NACP and nCaF_2_ into a low-shrinkage-stress nanocomposite holds the potential to enhance the longevity of dental restorations. This innovation has the capability to reduce polymerization stress biofilm formation and protect tooth structures by encouraging remineralization due to the substantial release of Ca, P, and F ions. Further research is required; however, to explore the long-term effects of the antibiofilm and remineralization properties of low-shrinkage-stress nanocomposite, integrating a combination of nCaF_2_, NACP, and DMADDM is required. Additionally, it is essential to investigate the marginal seal of this innovative low-shrinkage-stress nanocomposite in comparison to commercially available composites.

## 5. Conclusions

This study developed an innovative low-shrinkage-stress nanocomposite with antibiofilm and remineralization capabilities. The new formulations, which incorporated DMADDM with NACP, nCaF_2_, or a combination of both, demonstrated a potent antibiofilm effect while maintaining excellent ion release. These formulations achieved a reduction of 3 to 4 logs in saliva-derived biofilm CFU. Furthermore, there was a significant decrease in both biofilm metabolic activity and lactic acid production. Moreover, the low-shrinkage-stress nanocomposite exhibited a significant release of Ca, P, and F ions. The new bioactive low-shrinkage-stress nanocomposite has promise in various restorative dentistry applications for reducing cariogenic biofilms, protecting tooth structures, and improving marginal integrity by reducing shrinkage stress.

## Data Availability

The data presented in this study are available on request from the corresponding author.
